# A Machine-Learning-Based Medical Imaging Fast Recognition of Injury Mechanism for Athletes of Winter Sports

**DOI:** 10.3389/fpubh.2022.842452

**Published:** 2022-03-17

**Authors:** Peihua Liu, Nan Yue, Jiandong Chen

**Affiliations:** College of Physical Education, Beihua University, Jilin, China

**Keywords:** winter sports, CV model, SVM, medical image segmentation, medical image registration

## Abstract

The Beijing 2022 Winter Olympics will begin soon, which is mainly focused on winter sports. Athletes from different countries will arrive in Beijing one after another for training and competition. The health protection of athletes of winter sports is very important in training and competition. The occurrence of sports injury is characterized by multiple factors, uncertainty, and accidents. This paper mainly pays attention to the head injury with the highest severity. Athletes' high safety awareness is a part of reducing injury, but safety awareness cannot effectively reduce the occurrence of injury in competition, and timely treatment of injured athletes is particularly important. After athletes are injured, a telemedicine image acquisition system can be built, so that medical experts can identify athletes' injuries in time and provide the basis for further diagnosis and treatment. In order to improve the accuracy of medical image processing, a C-support vector machine (SVM) medical image segmentation method combining the Chan-Vese (CV) model and SVM is proposed in this paper. After segmentation, the edge and detail features of the image are more prominent, which meet the requirements of high precision for medical image segmentation. Meanwhile, a high-precision registration algorithm of brain functional time-series images based on machine learning (ML) is proposed, and the automatic optimization of high-precision registration of brain function time-series images is performed by ML algorithm. The experimental results show that the proposed algorithm has higher segmentation accuracy above 80% and less registration time below 40 ms, which can provide a reference for doctors to quickly identify the injury and shorten the time.

## Introduction

Beijing 2022 Winter Olympics is coming, and medical security is an indispensable part of the security work of the Winter Olympics (1). The Winter Olympic Games are mainly divided into skating and skiing (2). Among them, speed skating, ice hockey, alpine skiing, ski jumping, and other events are very easy to cause sports injuries in the process of competition and training. Sports injuries seriously limit the competition performance and level of winter sports and even affect the athletes' sports career (3–5). Emergency plans should be made for the situation of injuries, so as to coordinate with the activities of clinical medical treatment and rehabilitation treatment caused by sports injuries and ensure that the Beijing Winter Olympics will be held successfully. The application of high-tech will make the medical security of the Winter Olympics more intelligent and efficient. The 5G telemedicine technology allows ambulance to monitor the heartbeat in real-time and transmit the patient's physical signs to the medical center in real-time. In addition, telemedicine image acquisition can timely transmit the condition of critically ill patients to designated hospitals of the Winter Olympics, making real-time expert consultation possible and gaining time for rapid treatment (6–8).

Any sport has certain danger and may cause physical injury, and winter sports are no exception, so it is very important to protect the health of athletes. According to relevant studies, ice hockey, freestyle skiing, speed skating, figure skating, and other winter sports have a high injury rate (9), while knee joint, ankle joint, thighs, and shins are the main injury sites (10). The types of sports injuries are various, and the common ones are joint sprain, skin abrasion, contusion, fall, and collision of individual parts of the body. The main factors leading to injury are fierce confrontation, unreasonable collision, fatigue, lack of self-protection consciousness, and movement technical error. The major injury in winter sports is the injury of the head and spinal cord, and its severity is self-evident. Athletes' awareness of safety is part of injury reduction, and timely treatment of injured athletes is even more important. After an athlete is injured, the telemedicine image acquisition can be constructed to timely identify the injury of the athletes and provide a basis for further diagnosis and treatment (11, 12).

Different from ordinary images, medical images reveal the internal anatomical image rather than the surface features of the image. For different parts of medical images that need to be examined, the required image size is also different, so how to segment the image correctly becomes the basis of image analysis. Generally, medical images are very complex and highly inconsistent, and there is no room for any error in the state of patients, which requires high segmentation accuracy of medical images (13, 14). Since entering the field of support vector machine (SVM) has attracted extensive attention and developed comprehensively (15). It has become a standard tool in the field of machine learning (ML) and data mining. In recent years, it has successively proposed algorithms and applications for training SVM. At the same time, it has been verified in many practical problems that SVM has good performance, such as face recognition and fingerprint recognition.

In medical image analysis, several images of the same patient are often put together for analysis, so as to obtain comprehensive information of the patient from many aspects and improve the level of medical diagnosis and treatment. The same part of the body can produce different images based on different imaging principles, such as a slice of the human brain. Supposing that there are two known medical images, one (A) as a reference and the other (B) represents fluctuation. If there is a function transformation that B can find the only corresponding point in A under the related transformation, and their spatial positions are the same, then A and B are in image registration (16). In recent years, deep learning technology has exerted an important influence on the research of medical image processing methods and developed rapidly in the field of medical image registration.

The main contributions of this paper can be summarized as follows: (i) A C-SVM-based medical image segmentation method is proposed. (ii) The ML optimization process for medical image registration of brain functional time-series images is designed. The rest of this paper is organized as follows. Section Related Work reviews the related work. In sections Medical Image Segmentation and Medical Image Registration, medical image segmentation and medical image registration are studied, respectively. The experimental results are presented in section Experiment and Results Analysis. Finally, section Conclusions concludes this paper.

## Related Work

In recent years, many new methods have been proposed and applied to the field of image segmentation and achieved good results. However, in medical image segmentation, given the limitations of training samples, the traditional image segmentation methods, especially the methods of image segmentation based on single or single feature information, cannot meet the actual needs, so the development of medical image segmentation algorithm is still in the forefront of this field. In An and Liu (17), a multi-level boundary cognition and self-attention-based deep learning image segmentation algorithm was proposed. In (18), the authors proposed fuzzy entropy and Shannon entropy maximum threshold segmentation methods. In Zhang and Meng (19), the authors studied the deep learning-based medical image pixel block feature learning method. In Huang et al. (20), a novel segmentation framework was proposed to integrate the anatomical prior loss of medical images into the deep learning model. In Xie et al. (21), a novel general framework was proposed to improve the accuracy of deep convolutional neural networks in medical image segmentation. In Yang and Jia (22), an efficient and robust active contour model was proposed to segment and rectify images simultaneously. In Cunha et al. (23), the authors used Kohonen neural network for medical image segmentation, so that the neural network itself adopted unsupervised learning in the training process. In Ma et al. (24), the authors proposed a self-adaptive local fitting active contour model to realize robust segmentation of area-of-interest. Aiming at the problem that it was difficult to find and extract effective features in medical image segmentation, in An and Liu (25), a feedback mechanism convolution neural network-based medical image segmentation algorithm was proposed.

With the development of medical image processing technology, the use of image information processing technology for brain functional time sequence image analysis can improve the ability of pathological analysis and diagnosis of brain function, the study of high-precision registration of brain function time sequence image has attracted great attention. The advantage of ML is that it has a strong generalization ability. No matter what type of image, ML can find the required information and effectively improve the ability of image target recognition and image registration. In (26), a fast learning framework-based deformable and pairwise medical image, registration method was proposed. In Blendowski et al. (27), an end-to-end trainable, weakly-supervised deep learning-based feature extraction was studied. In Eppenhof et al. (28), a 3D Convolutional Neural Networks (3DCNN)-based deformable registration approach was proposed. In Lan et al. (29), a novel non-rigid registration method for medical images was proposed to realize the accurate positioning of images. In Pan and Zhang (30), a Renyi quadratic mutual information-based medical image registration method was proposed. In Alam et al. (31), a medical image registration method based on automatic features was proposed, which used the common sub-region of interest to realize rigid registration and deformable registration. In Sokooti et al. (32), a method of automatic quantitative prediction of registration error was proposed and applied to chest CT scanning. In Saygili (33), a target registration algorithm based on block matching on three orthogonal planes rather than three-dimensional space was proposed to estimate the target registration error with less computation. In Ma et al. (34), an unsupervised deformable image registration network was proposed for 3D medical images. In Gui et al. (35), the hybrid differential search algorithm was presented to optimize the cross-cumulative residual entropy algorithm for medical image registration.

## Medical Image Segmentation

Aiming at the head injury of athletes of winter sports during the Winter Olympics, this paper studies the medical imaging method based on ML, mainly focusing on medical image segmentation and medical image registration. These two aspects play a very important role in constructing the acquisition of telemedicine images.

**Figure 1:**
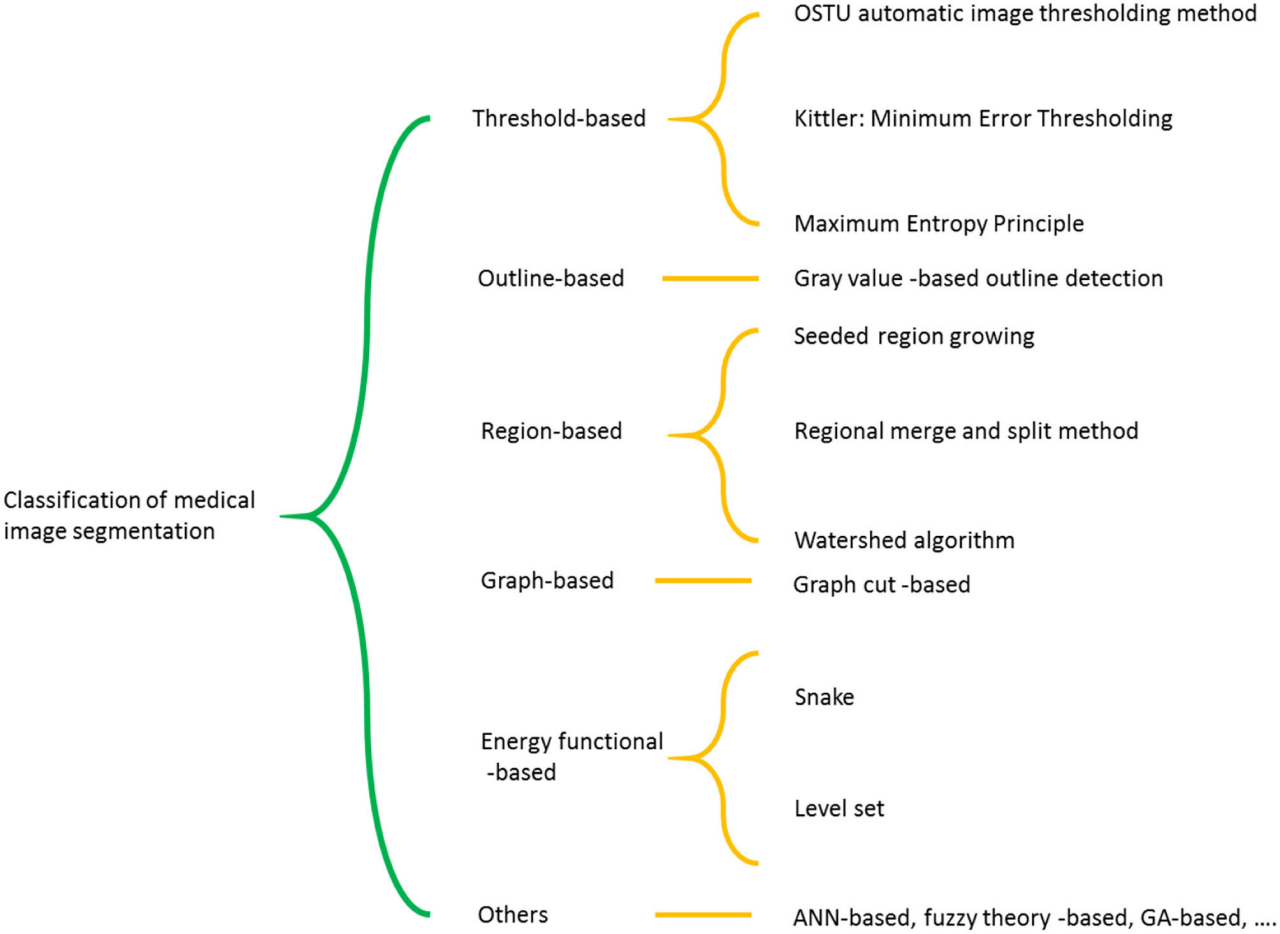
Classification of medical image segmentation schemes.

In MRI, normal brain tissue consists of white matter, gray matter, and cerebrospinal fluid. The purpose of image segmentation is to separate the brain tissue from the skull and scalp and then divide it into white matter, gray matter, and cerebrospinal fluid. In this paper, according to the good classification performance of the SVM method and combined with Chan-Vese (CV) model, a new method of C-SVM is proposed for medical image segmentation to study the brain MR image segmentation, and the target outline contour and individual data are successfully obtained. Classification of medical image segmentation schemes is shown in Figure 1.

### CV Model

Chan-Vese model (36) is a segmentation model based on regional information, which is actually an optimization of the simplified Mumford-Shah (MS) model (37). MS model does not use the edge detection function based on gradient but directly uses all the gray information inside and outside the active contour. Therefore, the MS model can better process image segmentation problems, such as strong noise edge blurring and has achieved success in the segmentation of gradient meaningless or edge blurring images. The MS model can detect the internal contour, which is not sensitive to noise. In recent years, image segmentation has been widely used in the field.

When MS functional is used for processing, the gray value of each homogeneous region in the medical image is set as a constant, so the fitting energy function of a simplified CV based on the MS model is defined as follows.


(1) $$\begin{array}{r} f\;(C,g_{0},g_{1})=\alpha Length\;(C)+\beta Area\;(inside\;(C))+\gamma_{1}\\ \int |u-g_{0}{|}^{2}dxdy+\gamma_{2}\int |u-g_{1}{|}^{2}dxdy \end{array}$$

Where α, β, γ_1_, and γ_2_ are non-negative constants. α and β are randomly set between 0 and 1. γ_1_ and γ_2_ are usually set 0.3 and 0.7, respectively (38). $\gamma_{1}\int |u-g_{0}{|}^{2}dxdy$ and $\gamma_{2}\int |u-g_{1}{|}^{2}dxdy$ represent the integration of the inside and outside regions of contour *C* respectively. *g*_0_ and *g*_1_ are the gray mean values inside and outside contour *C* respectively. *u* represents the gray value of each homogeneous region.

It can be seen from Equation (1) that the CV model can only divide the image into inside and outside regions of the fitting region, while medical images, such as human brain MR images, are multi-target complex images, which is difficult to achieve the purpose by using CV model alone. The application of SVM in image multi-classification makes up for this defect. Therefore, this paper proposes a new method of using SVM to segment the image after CV model segmentation.

The selection of SVM kernel function and parameters directly affects the classification results. Different kernel function classifiers have different performances, and the Gaussian kernel function used in this method is defined as follows.


(2) $$\begin{array}{l} k\;(x,x^{\prime })=e^{-\frac{||x-x^{\prime }|{|}^{2}}{2\sigma^{2}}} \end{array}$$

where *x* is any point, *x*′ is the center point, and σ is the width of the Gaussian distribution.

### Feature Extraction

In addition to the selection of kernel function, the effect of medical image segmentation also depends on the extraction of image features. The features of the medical image are generally represented by the gray-level co-occurrence matrix (GLCM), which can reflect the comprehensive information of the gray level in the orientation, adjacent interval, and amplitude change of medical images, from which they further extract the features that can describe the image texture.

The matrix [*G*(*i, j, d*, θ)] with orientation θ and interval *d* is defined as GLCM, and the dimension of the matrix is equal to the image gray level. It is assumed that the gray value of point (*m, n*) is *i*, and the gray value of point (*x, y*) is *j*, and *P* (*i, j, d*, θ) represents the occurrence frequency of elements in the *i*th row and *j*th column of the matrix. The relationship between these two points can be expressed as follows.


(3) $$\begin{array}{l} \;(x,y)=\;(m,n)+\;(d\cos\theta ,d\sin\theta ) \end{array}$$

In Equation (3), interval *d* is Euclidean distance. In this paper, the value of *d* is set as 1 (39), and θ is set to 0, 45, 90, and 135 degrees.

A series of features describing image texture can be extracted from GLCM. In order to make the expression more concise, the following co-occurrence matrix expression omits the interval *d* and orientation θ. Let *p* (*i, j*) be the GLCM with interval *d* and orientation θ generated by sub-images, where *i* and *j* represent gray level, then five statistics reflecting texture features are extracted as follows.

1. Angular Second Moment (ASM)


(4) $$\begin{array}{l} ASM=\underset{i}{\sum }\underset{i}{\sum }{p\;(i,j)}^{2} \end{array}$$

Angular second moment reflects the image gray distribution uniformity and texture fineness. If the element values of GLCM are similar, the energy is small and the texture is delicate. If some of these values are large and others are small, the energy value is large. Large energy values indicate a more uniform and regular texture pattern.

2. Contrast


(5) $$\begin{array}{l} Contrast=\underset{i}{\sum }\underset{i}{\sum }{p\;(i,j)\;(i-j)}^{2} \end{array}$$

The contrast reflects the clarity of the medical image and the depth of the groove of the texture. The image with deep grooves and large contrast texture is more clear. On the contrary, for the image with small contrast, the grooves are shallow and the effect is fuzzy.

3. Correlation


(6) $$\begin{array}{l} \text{Correlation}=\frac{\sum_{i}\sum_{i}\;(i\cdot j)\;p\;(i,j)-\mu_{x}\cdot \mu_{y}}{\sigma_{x}{\cdot \sigma }_{y}} \end{array}$$

Let $p_{x}\left(i\right)=\overset{I_{g}}{\underset{j=1}{\sum }}p\left(i,j\right)$ and $p_{y}\left(j\right)=\overset{I_{g}}{\underset{i=1}{\sum }}p\left(i,j\right)$. μ_*x*_, μ_*y*_, σ_*x*_, and σ_*y*_ are the mean values, and SD of *p*_*x*_ (*i*) and *p*_*y*_ (*j*), respectively. *I*_*g*_ is the gray level of medical images. The correlation is used to measure the similarity degree of medical image gray level in a row or column direction, so the value reflects the local gray-level correlation, and the larger the value is, the greater the correlation is.

4. Variance


(7) $$\begin{array}{l} \text{Variance}=\underset{i}{\sum }\underset{i}{\sum }\;(i\cdot j-\mu_{x}\cdot \mu_{y})\;p\;(i,j) \end{array}$$

The variance reflects the variation characteristics of the medical image gray scale.

5. Entropy


(8) $$\begin{array}{l} \text{Entropy}=-\underset{i}{\sum }\underset{i}{\sum }p\;(i,j)\log p\;(i,j) \end{array}$$

When all values in the co-occurrence matrix are equal or the pixel values show the maximum randomness, the entropy reaches the maximum. Therefore, entropy indicates the complexity of medical image gray distribution. The larger the entropy value is, the more complex the image is.

In the calculation of GLCM, the window size needs to be determined. In this paper, the brain MR medical image selects a window with a size of 7 × 7. In practical application, it is found that the classification ability of the algorithm is relatively low when the sample size is limited and the two types of samples are greatly different. Therefore, this paper adopts the C-SVM segmentation method that combines SVM and CV model.

## Medical Image Registration

MRI can make many non-invasive observations and measurements of the human brain and can accurately reveal the whole process of physiological and pathological activities of the human brain. Physical and physiological head movements will inevitably occur in the process of MRI. Although these head movements are small, they will make it difficult to capture hemodynamic signals. Therefore, in brain MRI, head movements of only tens of microns will cause temporal differences in brain functional series images.

### Image Visual Feature Extraction

In this paper, a high-precision registration algorithm of brain functional time-series images based on ML is proposed. At first, the brain functional time-series images are acquired, and the 3D visual information acquisition model of brain functional time-series images is constructed. Then the visual feature extraction and information enhancement of brain functional time-series images are realized. Based on this, the optimal design of high-precision registration of brain functional time-series image is carried out. The block matching of spatial visual features of brain functional time-series images is performed by using multifractal technology, and the correlation feature matching is carried out for details of different resolutions. The multi-level and multi-direction decomposition method is adopted to obtain the spatial similarity feature of brain functional time-series images, which can be defined as follows.


(9) $$\begin{array}{l} B\;(k)=\phi B\;(\varepsilon -1)+d\;(k) \end{array}$$

where φ represents the correlation feature matching coefficient, *B* represents the correlation degree of brain functional time-series images, and *d* (*k*) represents the multi-level and multi-direction decomposition function.

The spatial visual feature distribution values of the brain functional time-series images are extracted, and the adaptive fusion and optimal segmentation of the brain functional time-series images are combined with the sparse structure feature decomposition method. According to the feature segmentation results, multi-layer spatial structure feature registration and fusion clustering of brain functional time-series images are performed to realize information enhancement and visual information feature extraction of brain functional time-series images.

Given the above, the visual feature extraction of brain functional time-series images is realized, and then ML and optimization are performed according to the feature extraction results.

### Machine-Learning Algorithm Optimization

A 4 × 4 sub-block segmentation model is used for space visual rotation and feature extraction of brain functional time-series images. Adaptive optimization is carried out in the 3D distribution region of brain functional time-series images. The block feature matching method is used to sample the associated frames of the original brain functional time-series images.

In region *A*_*h*_, the ML optimization process for image registration of brain functional time-series images is designed, and the specific process is as follows

Step 1: The point *h* is randomly selected as the fuzzy pixel in the unlabeled registration output sample set of brain functional time-series images to be tested.
Step 2: Update the coordinates of point *h*. *h* ← *h* + *M*, and *M* is the threshold of the regional gradient.
Step 3: Repeat step 1 to step 2 until the condition *M* < τ is met. Take *h* at this time as the center point of the cluster, mark the points in the region *A*_*h*_, and add them into the cluster.
Step 4: If the central point coordinates of all clusters remain unchanged, it means that the result converges and the calculation stops. If the distance between the center of the current cluster *c*_*m*+1_ and the center of other existing cluster *c*_*m*_ is less than the threshold *M*, then the two clusters need to be combined. Otherwise, *c*_*m*+1_ is taken as a new cluster.
Step 5: Repeat step 2 to step 4 until all points are marked and fuzzy pixel set *F* is obtained.
Step 6: The fuzzy correlation feature detection method is used to extract the gray features of the brain functional time-series images in the above set. According to the feature extraction results, the Adaboost algorithm (38) is used to automatically optimize the high-precision registration of the brain functional time-series images, and the output is defined as follows.

(10) $$\begin{array}{l} \{\begin{array}{c} x=T\sin\gamma \cos\varphi \\ y=T\sin\gamma \sin\varphi \\ z=T\cos\gamma \end{array}\;F\leq 0 \end{array}$$

where γ represents the edge brightness of the brain functional time-series image, and *T* represents the template matching coefficient of the brain functional time-series image.

## Experiment and Results Analysis

### Setup

#### Image Segmentation

In order to verify the segmentation effect of the proposed C-SVM model, the McConnell brain Imaging Center online image library is used for segmentation experiments. The experimental data is T1-weighted MRI images with 2% noise, 30% gray-level non-uniformity, and slice thickness of 2 mm. The kernel function is the Gaussian radial kernel function (σ = 0.5, *C*= 1,000). The number of training samples is 1,500, and the window size is 7 × 7. The brain tissues, such as gray matter, white matter, and cerebrospinal fluid, are segmented in different training areas and the experimental results are given. In medical image segmentation, multi-level boundary cognition and self-attention (MLBC-SA) (17), fuzzy entropy and maximum entropy (FE-ME) (18), and deep learning-based medical image segmentation (DL-MIS) (19) are used for comparison.

#### Image Registration

In medical image segmentation, VoxelMorph (26), end-to-end weakly-supervised (EEWS) (27), and 3DCNN-deformable registration (3DCNN-DR) (28) are used for comparison. In addition, 800 groups of brain functional images of McConnell brain Imaging Center online image library are used, and 100 pixels are randomly sampled from each functional brain image. The iterative step number of ML is 100, the step size of adaptive iteration is 10, the gradient descent rate of feature registration is 0.45, and the characteristic quantization coefficient of edge evolution is 0.62.

### Comparison Analysis

#### Image Segmentation

**Figure 2:**
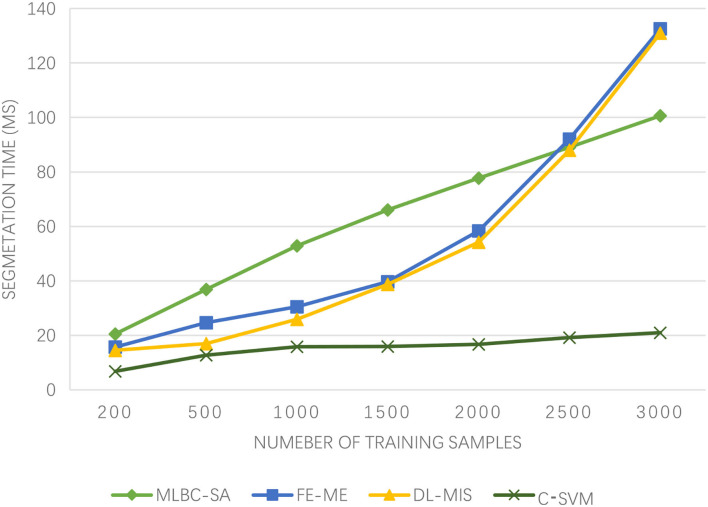
Experimental results of segmentation time.

As can be seen from Figure 2, the medical image segmentation time of the algorithm proposed in this paper is always the lowest as the increasing number of training samples, basically within 20 ms, and the growth is relatively gentle. The medical image segmentation time trend of the FE-ME algorithm and DL-MIS algorithm is consistent and increases rapidly. In contrast, although the medical image segmentation time of the MLBC-SA algorithm is also increasing, it is relatively flat. More surprisingly, after the number of training samples reaches 2,500, the segmentation time of the MLBC-SA algorithm is even lower than that of the FE-ME algorithm and DL-MIS algorithm. On the whole, the medical image segmentation time of the algorithm proposed in this paper is very low, which is significant for the medical imaging of athletes of winter sports after injury. A lower segmentation time means a lower risk, which provides strong support for the treatment of athletes of winter sports in terms of time.

**Figure 3:**
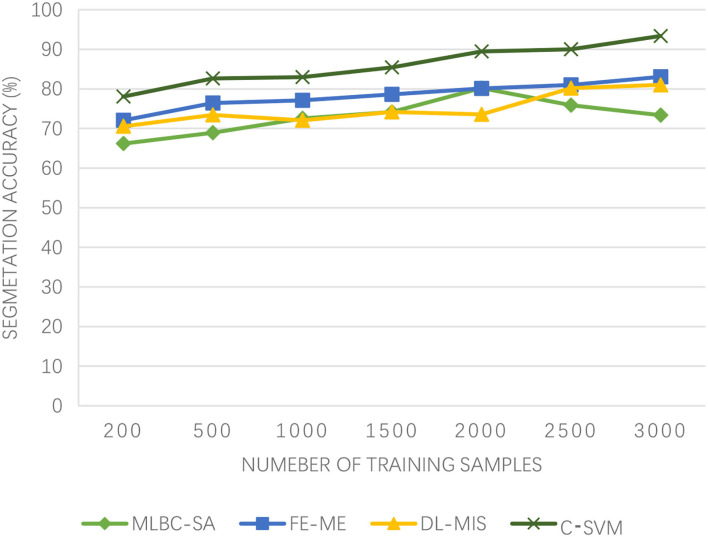
Experimental results of segmentation accuracy.

As can be seen from Figure 3, the segmentation accuracy of the proposed algorithm has been on the rise and has been very high, basically above 80%. However, with the increasing number of training samples, the segmentation accuracy of DL-MIS fluctuates around 75%, which is not very stable. Although the segmentation accuracy of the MLBC-SA algorithm has a good upward trend when the number of samples is small, it declines when the number of samples reaches 2,000. On the other hand, although the FE-ME algorithm has been showing an upward trend and is relatively slow. The Winter Olympics is dominated by winter sports, and while athletes will be careful, there will be the emergencies. Once an accident occurs, the brain injury is very fatal. Even the slightest difference in the accuracy of MR image segmentation will lead to the doctor's wrong judgment. Therefore, the algorithm proposed in this paper can ensure the high accuracy of brain MR image segmentation and protect the athletes of winter sports.

#### Image Registration

**Figure 4:**
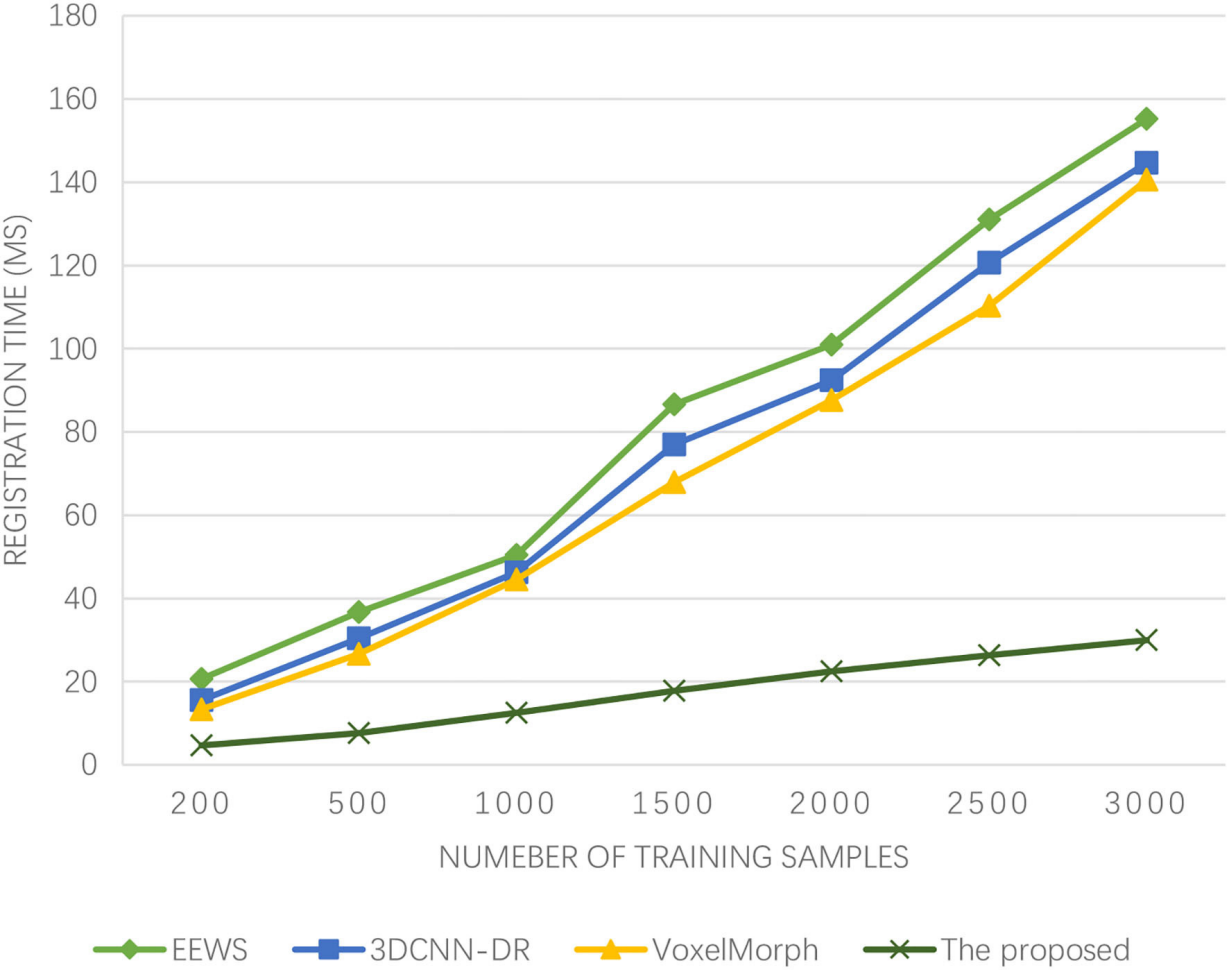
Experimental results of registration time.

It can be seen from Figure 4 that the registration time of the four algorithms increases with the increasing number of samples. The registration time of EEWS is 20.67–155.20 ms, 3DCNN-DR is 15.54–144.66 ms, and VoxelMorph is 13.34–140.56 ms. However, the registration time cost of the proposed algorithm is <30 ms, and the registration time is the shortest. The image registration efficiency is the highest, and with the increasing number of samples, the registration time of the proposed algorithm rises slowly and goes to stable. If there is brain injury in winter sports, it is more serious. The low registration time wins the most precious time for the treatment of athletes and also provides time support for doctors.

**Figure 5:**
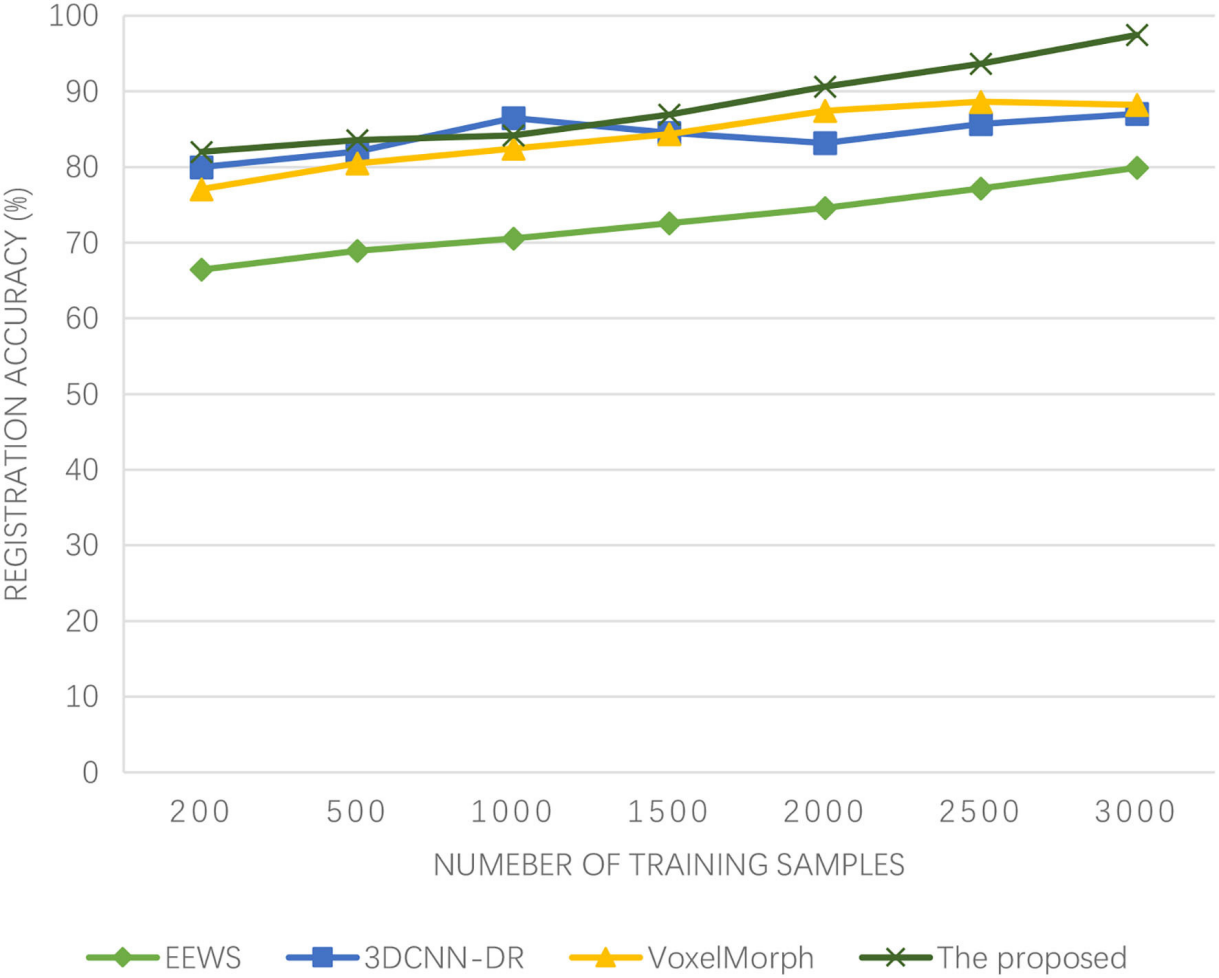
Experimental results of registration accuracy.

As can be seen from Figure 5, the algorithm proposed in this paper has been improving the registration accuracy of medical images with the increasing number of training samples. When the number of training samples reaches 2,000, the registration accuracy has exceeded 90%. The 3DCNN-DR algorithm also has a high registration accuracy at the beginning, but when the number of training samples reaches 1,000, the registration accuracy actually decreases. Similarly, the registration accuracy of the VoxelMorph algorithm also shows a declining trend when the number of training samples reaches 2,500. Although the registration accuracy of the EEWS algorithm has been improved, even when the number of training samples reaches 3,000, its registration accuracy does not exceed 80%. The algorithm presented in this paper has high registration accuracy and can effectively achieve high-precision brain functional time-series image registration. This is because Adaboost is a summation model, and each model is based on the error rate of the previous model. Excessive attention is paid to the misclassified samples, while attention is reduced to the correctly classified samples. After successive iterations, a relatively good model can be obtained. Adaboost ML algorithm makes good use of weak classifiers to cascade, and different classification algorithms can be used as weak classifiers. When simple classifiers are used, the calculated results are understandable, and the construction of weak classifiers is extremely simple. The most important thing is that overfitting is not easy to happen in the Adaboost ML algorithm. The accuracy of medical image registration is how to quickly register the image when the athletes of winter sports are physically injured, especially when the brain of athletes is injured, which reflects the superiority of the algorithm, and this is the ability that should be possessed in the emergency treatment of athletes of winter sports.

## Conclusions

The 2022 Winter Olympics will be held in Beijing. The health protection of athletes of winter sports is very important. In training or competition, athletes' injuries are inevitable, so it is imperative to quickly identify athletes' injuries. This paper uses the ML method to build a telemedicine medical image acquisition and studies the two important fields of medical image segmentation and medical image registration. A new method of using SVM is proposed to segment the image after CV model segmentation. A series of features describing image texture can be extracted from GLCM and five statistics reflecting texture features are extracted, which are ASM, contrast, correlation, variance, and entropy. In medical image registration, a high-precision registration algorithm of brain functional time-series images based on ML is proposed. The multi-level and multi-direction decomposition method is adopted to obtain the spatial similarity feature of brain functional time-series images, and the ML optimization process for image registration of brain functional time-series images is designed. The experimental results reveal that the proposed algorithm outperforms benchmarks in terms of segmentation accuracy, segmentation time, registration accuracy, and registration time. Through the comparison of four metrics, it can be seen that the algorithm proposed in this paper has improved greatly in both the accuracy and time of medical image segmentation and medical image registration in telemedicine image acquisition after injury of athletes of winter sports. This also saves time in treating athletes and reduces the risk of further illness.

The future and current pieces of work include: (1) introducing graph cutting, confidence propagation, and other algorithms to realize the optimal energy solution and discussing how to optimize the energy minimization model to improve the segmentation efficiency of the algorithm; (2) explore other algorithms, such as deep learning method, which are applied to medical image segmentation such as cell and retina; (3) in image registration, the model lacks some flexibility in training, and the subsequent work will consider designing a method that can adjust the weight adaptively through the internal information of the image.

## Data Availability Statement

The raw data supporting the conclusions of this article will be made available by the authors, without undue reservation.

## Author Contributions

PL contributed to writing. NY contributed to methods. JC contributed to data analysis. All authors contributed to the article and approved the submitted version.

## Funding

This work was supported by Social Science Project of Jilin Province Education Department in 13th Five-Year (No. JJKH20180395SK), Youth Teaching Reform in Beihua University in 2019 (No. XJQN2019032), and Key Teaching and Research Projects in Beihua University in 2021 (No. XJZD2021028).

## Conflict of Interest

The authors declare that the research was conducted in the absence of any commercial or financial relationships that could be construed as a potential conflict of interest.

## Publisher's Note

All claims expressed in this article are solely those of the authors and do not necessarily represent those of their affiliated organizations, or those of the publisher, the editors and the reviewers. Any product that may be evaluated in this article, or claim that may be made by its manufacturer, is not guaranteed or endorsed by the publisher.
